# The Extracytoplasmic Stress Factor, σ^E^, Is Required to Maintain Cell Envelope Integrity in *Escherichia coli*

**DOI:** 10.1371/journal.pone.0001573

**Published:** 2008-02-06

**Authors:** Jennifer D. Hayden, Sarah E. Ades

**Affiliations:** Department of Biochemistry and Molecular Biology, The Pennsylvania State University, University Park, Pennsylvania, United States of America; University of Massachusetts, United States of America

## Abstract

Extracytoplasmic function or ECF sigma factors are the most abundant class of alternative sigma factors in bacteria. Members of the *rpoE* subclass of ECF sigma factors are implicated in sensing stress in the cell envelope of Gram-negative bacteria and are required for virulence in many pathogens. The best-studied member of this family is *rpoE* from *Escherichia coli*, encoding the σ^E^ protein. σ^E^ has been well studied for its role in combating extracytoplasmic stress, and the members of its regulon have been largely defined. σ^E^ is required for viability of *E. coli*, yet none of the studies to date explain why σ^E^ is essential in seemingly unstressed cells. In this work we investigate the essential role of σ^E^ in *E. coli* by analyzing the phenotypes associated with loss of σ^E^ activity and isolating suppressors that allow cells to live in the absence of σ^E^. We demonstrate that when σ^E^ is inhibited, cell envelope stress increases and envelope integrity is lost. Many cells lyse and some develop blebs containing cytoplasmic material along their sides. To better understand the connection between transcription by σ^E^ and cell envelope integrity, we identified two multicopy suppressors of the essentiality of σ^E^, *ptsN* and *yhbW*. *yhbW* is a gene of unknown function, while *ptsN* is a member of the σ^E^ regulon. Overexpression of *ptsN* lowers the basal level of multiple envelope stress responses, but not that of a cytoplasmic stress response. Our results are consistent with a model in which overexpression of *ptsN* reduces stress in the cell envelope, thereby promoting survival in the absence of σ^E^.

## Introduction

All organisms have stress responses that allow them to sense and respond to damaging conditions by altering gene expression. An additional level of complexity is introduced when the inducing signal is sensed on one side of a membrane and that information must be communicated across the membrane for a response to be generated. In Gram-negative bacteria this intercompartmental signaling is required to maintain the cell envelope, which consists of the inner and outer membranes, periplasmic space, and peptidoglycan layer [1].

The cell envelope is a complex, dynamic compartment that is crucial for the survival of the cell. It is not a static structure and can be remodeled in response to environmental conditions. The chemical environment of the cell envelope is distinct from that of the cytoplasm. The envelope lacks ATP, is oxidizing, and can be subject to fluctuations in ionic strength due to passive diffusion of small molecules through outer membrane porins [2]. As such, Gram-negative bacteria possess stress responses that are uniquely targeted to the cell envelope. These stress responses include the CpxAR (Cpx), BaeRS (Bae), and Rcs phosphorelays, the response governed by the alternative sigma factor σ^E^, and the phage shock (PSP) response [3]–[6]. Each of these responses is activated following perturbation of particular components of the envelope.

Although stress responses are important for reacting to damaging conditions, many stress proteins also play important roles in basic cellular physiology. This is particularly true for the σ^E^-dependent response in *E. coli*, as the *rpoE* gene, which encodes σ^E^, is essential for viability [7]. Despite the wealth of information about the role of σ^E^ in response to cell envelope stress and the identification of the σ^E^ regulon, the essential role of σ^E^ is still unclear [8], [9]. One suppressor of Δ*rpoE* lethality, a deletion of the *ydcQ* gene, has been identified [10]. However, the function of this gene is not well understood and it is not clear how it suppresses the essentiality of *rpoE*.

**Figure 1:**
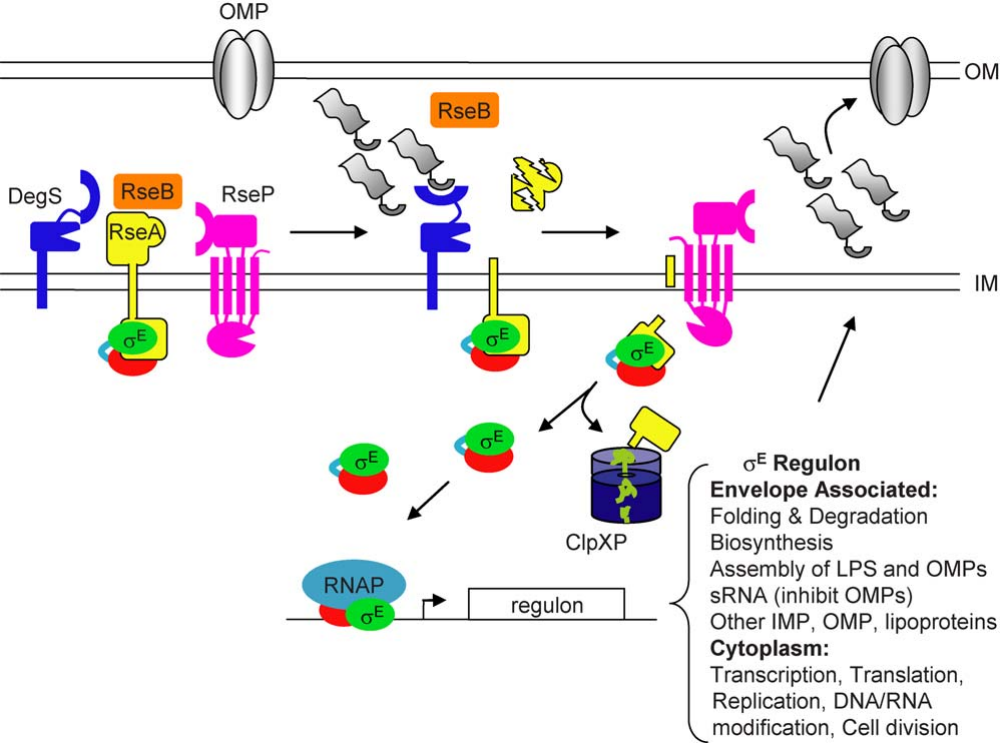
The σ^E^-dependent extracytoplasmic stress response. σ^E^ is held at the membrane by the antisigma factor RseA. RseB binds to the periplasmic domain of RseA and protects RseA from proteolysis. Unfolded OMPs activate the protease DegS, which cleaves the periplasmic domain of RseA. The partially degraded RseA is now a substrate for RseP. RseP cleaves RseA further, releasing the cytoplasmic domain of RseA bound to σ^E^. This remaining domain of RseA is degraded by ClpXP thereby freeing σ^E^ to interact with RNA polymerase and direct transcription of its regulon. The major classes of known genes in the σ^E^ regulon are indicated. The alarmone ppGpp and protein DksA can activate σ^E^-dependent transcription once σ^E^ is released from RseA, but are not shown for clarity. (IM inner membrane, OM outer membrane).

σ^E^ is activated by stresses that interfere with the folding of outer membrane proteins (OMPs) such as heat shock, overexpression of OMP genes, and mutations in genes encoding chaperones required for OMP folding [11]–[14]. In unstressed cells, σ^E^ activity is low because σ^E^ is sequestered at the inner membrane by the antisigma factor RseA (Fig. 1) [15], [16]. RseA is a single-pass inner membrane protein that binds to σ^E^ and prevents σ^E^ from interacting with RNA polymerase [15]–[17]. During envelope stress, RseA is degraded in response to unfolded porins by the sequential action of two inner membrane proteases, DegS and RseP, followed by the cytoplasmic protease ClpXP (Fig. 1) [18]–[22]. A periplasmic protein, RseB, binds to the periplasmic domain of RseA and enhances inhibition by RseA, protecting it from proteolysis (Fig. 1) [15], [16], [18], [23]. Continual degradation of RseA is required to provide the cell with sufficient free σ^E^ to support viability, and deletion of either *degS* or *rseP* is toxic due to the stabilization of RseA and consequent sequestration of σ^E^ [19], [20], [23]. σ^E^ can also be activated independently of the RseA-dependent stress-signaling pathway by the cytoplasmic alarmone ppGpp, whose levels change in response to nutrient availability [24].

As expected from its role in the stress response, the σ^E^ regulon includes genes encoding periplasmic foldases, proteases, and chaperones that aid in OMP folding. In addition, σ^E^ transcribes an array of biosynthetic enzymes that are involved in phospholipid, fatty acid, LPS, and membrane-derived oligosaccharide synthesis and transport, and a number of other cell envelope proteins including lipoproteins, inner membrane proteins, and envelope proteins of unknown function [9], [25], [26]. The σ^E^ regulon also includes several genes that encode major components of complexes responsible for ushering LPS and OMPs across the periplasm and properly assembling them in the outer membrane [27], [28]. Recently σ^E^ has been shown to transcribe several small RNAs that decrease the expression of OMPs, providing a mechanism to decrease the flux of OMPs to the envelope during times of stress [29]–[31]. In addition to the envelope-associated proteins, σ^E^-dependent promoters are found upstream of genes involved in cytoplasmic processes such as transcription, translation, DNA replication, and DNA/RNA modification [9], [25], [26].

The σ^E^ regulon contains a number of essential genes, and the simplest explanation for why σ^E^ is essential is that it is required to transcribe one or more of these genes. Alternatively, loss of expression of multiple non-essential genes dependent on σ^E^ for expression could be lethal. However, many genes in the σ^E^ regulon are also transcribed by σ^70^. Chromatin immoprecipitation followed by genomic microarray (ChIP-chip) experiments examining the location of σ^70^ on the *E. coli* chromosome suggest that approximately 40% of σ^E^-regulated promoters overlap with σ^70 ^promoters [32]. This number is likely to be an underestimation of the extent of dual regulation, since it does not include genes that also have σ^70 ^promoters farther away from the σ^E^ promoter [32]. As such, σ^E^ may be essential because genes in its regulon that are also transcribed by σ^70 ^are misregulated with lethal effect in the absence of σ^E^.

In this work we took two approaches to better understand the essential role of σ^E^. The first approach was to characterize the phenotypes of cells when σ^E^ activity was inhibited to gain insights into what cellular functions were impacted. We demonstrate that σ^E^ is required to maintain the integrity of the cell envelope; in the absence of σ^E^ the cells developed blebs along their sides and lysed. The second approach was to isolate multicopy suppressors that allow cells to live in the absence of σ^E^ activity. Two multicopy suppressors, *ptsN* and *yhbW*, were isolated that suppress both the lethality due to inhibition of σ^E^ activity and the essentiality of the *rpoE* gene. *ptsN* is a member of the σ^E^ regulon, while *yhbW* is not, and neither gene has previously been associated with cell envelope functions. Overexpression of *ptsN* lowered the basal activity of several envelope-sensing pathways, implying that it may suppress Δ*rpoE* lethality by reducing stress in the cell envelope.

## Results

### Inhibition of σ^E^ activity is toxic and increases envelope stress

**Figure 2:**
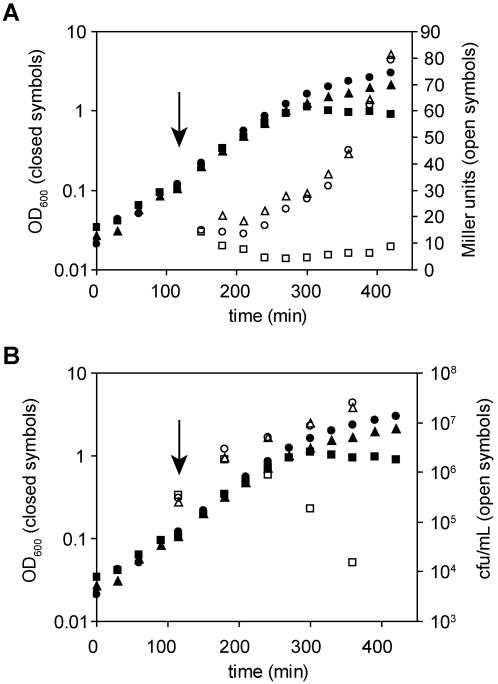
Inhibition of σ^E^ activity caused cell death. Strains SEA007 (squares, pRseAB), SEA008 (circles, pTrc99a vector control), and SEA4044 (triangles, pRseAD11H) were grown in LB at 30°C with shaking in a gyrotary waterbath. IPTG was added at OD_600_ ∼0.1 to induce overexpression of *rseA* and *rseB*, indicated by the arrow. (A) σ^E^ was inhibited by overexpression of *rseA* and *rseB*, but not *rseA*D11H and *rseB*, as shown by β-galactosidase activity measured from the σ^E^-dependent *rpoH*P3-*lacZ* reporter. Miller units (open symbols, right axis) and OD_600_ (closed symbols, left axis) are plotted at each time point. (B) Overexpression of *rseA* and *rseB*, but not *rseA*D11H and *rseB*, caused a reduction in the colony forming units and optical density. OD_600_ (closed symbols, left axis) and cfu/ml (open symbols, right axis) were measured throughout the growth curve. A representative experiment is shown in both panels.

To gain a better understanding of the role of a particular gene in cellular physiology, it is often informative to examine the phenotypes of a deletion mutant lacking the gene of interest. Because σ^E^ is encoded by an essential gene, it is not possible to examine the phenotype of a Δ*rpoE* strain. Therefore we examined the effects of loss of σ^E^ activity by inducing the overexpression of its inhibitors, *rseA* and *rseB*, encoded under the IPTG-inducible *trc* promoter on the pTrc99a plasmid, pRseAB. This method efficiently inhibits σ^E^ (Fig. 2A) by preventing its association with RNA polymerase [15]–[17]. Overexpression of *rseA* and *rseB* and the consequent sequestration of σ^E^ were lethal for the bacterium. In liquid culture, the cfu/ml began to decrease after 2 hours (∼2.5 generations) following the addition of IPTG and the optical density stopped increasing and began to decrease slightly within 3 hours (∼3.5 generation) (Fig. 2B and [15]). On solid media, the plating efficiency in the presence of IPTG was reduced by three orders of magnitude compared with non-inducing conditions (Table 1). To ensure that the observed phenotypes were due to inhibition of σ^E^ and not overproduction of RseA and RseB, we introduced a point mutation into the *rseA* gene on the plasmid encoding *rseA* and *rseB*, changing the aspartate residue at position 11 of RseA to histidine. This mutation abrogates the antisigma factor activity of RseA (Fig. 2A and [16]). When overproduced along with RseB, the RseAD11H protein reached similar steady-state levels as the wild-type protein, was properly localized to the inner membrane, did not inhibit σ^E^ activity, did not induce lysis, and did not reduce the plating efficiency (Fig. 2, Table 1, and data not shown).

**Table 1:** Plating efficiencies following inhibition of σ^E^

strain	plasmid	plating efficiency^a^
SEA008	pTrc99a	1.0 ± 0.3
SEA007	pRseAB	4.9 ± 2.7 × 10^−3^
SEA4044	pRseA^D11H^B	0.9 ± 0.1
SEA4256	pRseAB+pPtsN	0.9 ± 0.2
SEA4271	pRseAB+pYhbW	0.6 ± 0.3

a plating efficiency: cfu/ml with IPTG divided by cfu/ml no IPTG

**Figure 3:**
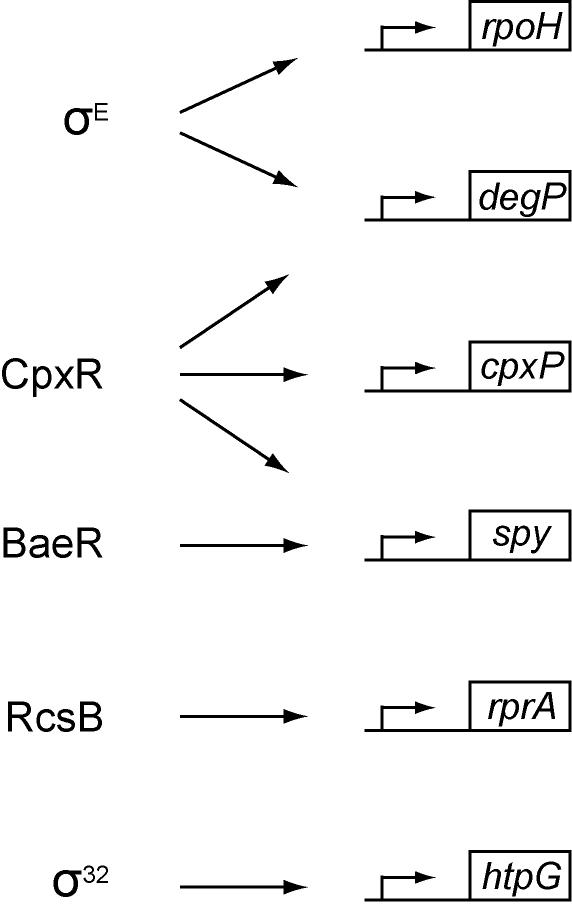
Diagram of stress regulators and their respective regulon members used as reporters in this work. All reporter constructs are present as *lacZ* fusions integrated as a single-copy in the chromosome. The *rpoH*P3-*lacZ* fusion is regulated by σ^E^. The *degP*-*lacZ* fusion is regulated by σ^E^ and CpxR. The *cpxP*-*lacZ* fusion is regulated by CpxR. The *spy*-*lacZ* fusion is regulated by CpxR and BaeR. The *rprA*-*lacZ* fusion is regulated by RcsB. The *htpG*-*lacZ* fusion is regulated by σ^32^. σ^E^, Cpx, Bae, and Rcs all monitor envelope stress, while σ^32 ^monitors cytoplasmic stress.

**Figure 4:**
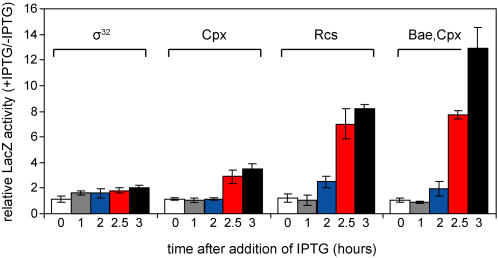
Envelope stress responses are activated following inhibition of σ^E^. β-galactosidase activity was measured from the *htpG*-*lacZ* (σ^32^, SEA4183), *cpxP*-*lacZ* (Cpx, SEA4177), *rprA*-*lacZ* (Rcs, SEA4190), and *spy*-*lacZ* (Cpx and Bae, SEA4187) reporters. The fold change in Miller units (M.U.) with and without *rseA* and *rseB* overexpression (M.U. with IPTG/M.U. no ITPG) is shown for samples taken at 0 (white), 1 (grey), 2 (blue), 2.5 (red), and 3 (black) hours after addition of IPTG.

Because σ^E^ is known to respond to envelope stress and direct the synthesis of many components of the cell envelope, it is likely that this compartment will be negatively impacted and envelope stress will increase when σ^E^ activity is blocked. We measured activation of reporter genes for the Cpx, Bae, and Rcs envelope stress responses (*cpxP*-*lacZ* activated by CpxR, *spy*-*lacZ* activated by BaeR and CpxR, *rprA*-*lacZ* activated by RcsB, Fig. 3). β-galactosidase activity from the envelope stress reporters increased by 2.5 hours following inhibition of σ^E^ (Fig. 4). Since activation of these stress responses could be due to a general increase in stress associated with cell death, we also monitored activation of the cytoplasmic stress response mediated by σ^32 ^using the σ^32^-regulated *htpG*-*lacZ* reporter fusion. σ^32^ activity increased relatively little (∼2-fold) following inhibition of σ^E^ compared with the larger increases seen for the envelope stress responses (3.5-fold for Cpx, 8-fold for Rcs, and 12-fold for the Bae/Cpx-dependent reporters). These results suggest that loss of σ^E^ activity does increase stress in the cell envelope, but does not increase cytoplasmic stress to the same extent.

### Loss of σ^E^ activity does not cause major changes in the composition of membranes

**Figure 5:**
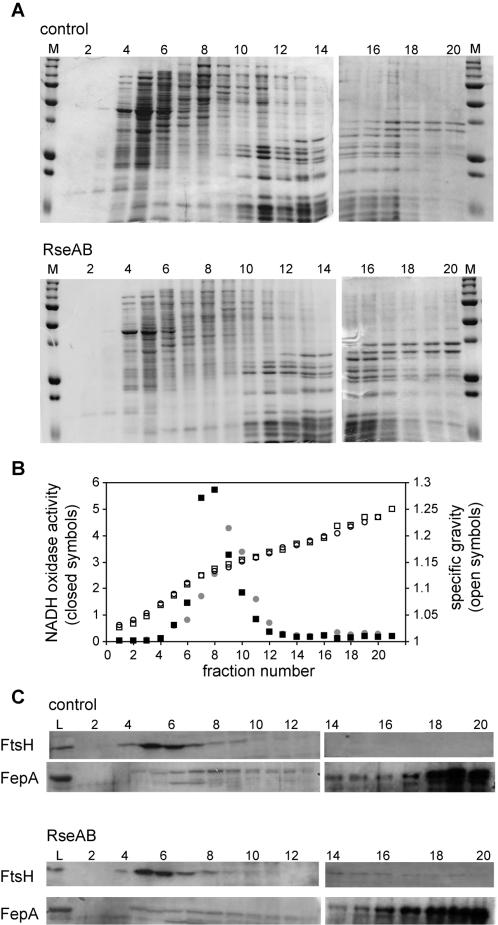
Fractionation of strain SEA007 lysates with and without inhibition of σ^E^ on sucrose density gradients. At 2.5 hours following inhibition of σ^E^, cells were harvested and lysates separated by discontinuous sucrose density gradient centrifugation. Lysates from comparable numbers of cells (as determined by OD_600_) were loaded on the gradients. (A) Coomassie-stained 12% SDS-polyacrylamide gels of gradient fractions from the control culture (top) and culture in which σ^E^ activity was inhibited (bottom). M denotes molecular weight markers. (B) β-NADH oxidase activity (closed symbols) and specific gravity (open symbols) of the fractions in (A), inhibition of σ^E^ (circles) and control (squares). (C) Western blots using polyclonal antibodies to detect FtsH (inner membrane) and FepA (outer membrane) in fractions from (A), control (top panels) and inhibition of σ^E^ (bottom panels). L denotes whole-cell lysates before fractionation.

Since inhibition of σ^E^ increases envelope stress and σ^E^ can transcribe genes that are localized to the inner and outer membranes, we next asked if the envelope membranes were altered. Samples were taken from cultures 2.5 hours following overexpression of *rseA* and *rseB*. This time point was chosen for analysis because the cell envelope stress responses were activated, the cfu/ml had started to decrease, and the morphological defects described below were evident. Cell lysates were fractionated by centrifugation through discontinuous sucrose density gradients and several assays were used to detect fractions containing inner and outer membrane components. In each of these assays the results from cultures in which σ^E^ activity was inhibited were similar to those from control cultures. No alterations in the overall protein content of the fractions were detected on Coomassie-stained SDS polyacrylamide gels (Fig. 5A). The inner and outer membrane fractions were well separated, as determined by western blots probed with antibodies to the inner membrane protein, FtsH, and the outer membrane protein, FepA (Fig. 5C). Fractions containing inner membrane were also identified using a β-NADH oxidase activity assay (Fig. 5B). Thus, loss of σ^E^ activity does not result in gross defects in the steady-state protein composition or density of the inner and outer membranes.

### Morphological changes following inhibition of σ^E^

**Figure 6:**
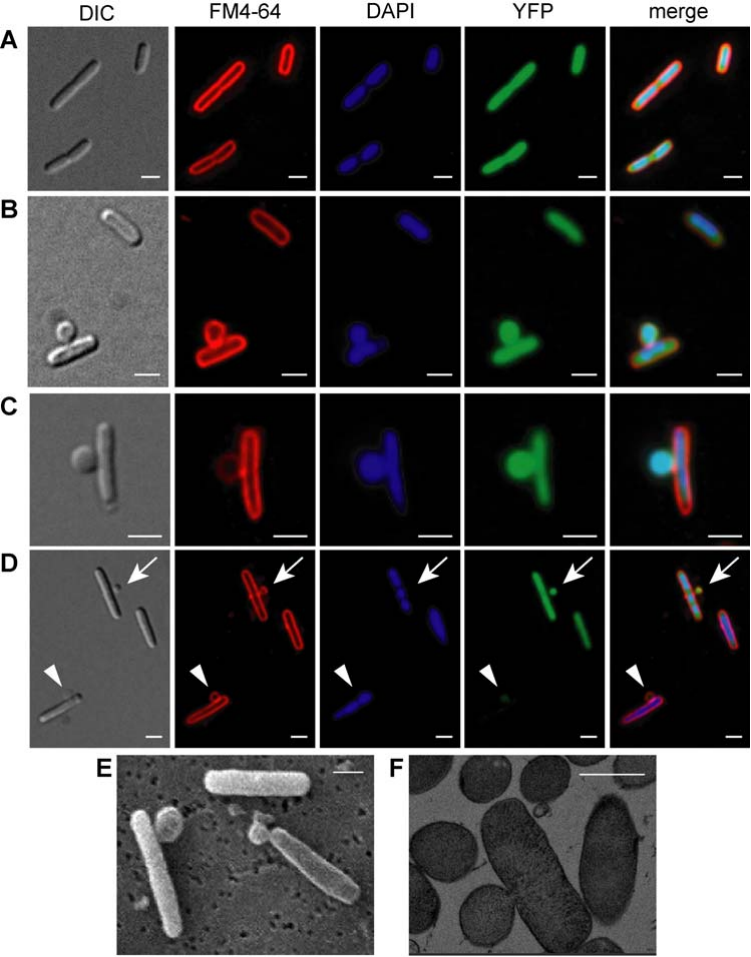
Cell envelope defects resulting from inhibition of σ^E^. Cultures of SEA007 and SEA008 were grown as in Fig. 2. Samples were taken approximately 2.5 hrs. after induction of *rseA* and *rseB* overexpression. (A–D) Images of live cells are shown using differential interference microscopy (DIC, column 1) and fluorescence microscopy following addition of FM4-64 to visualize membranes (red, column 2), DAPI to visualize DNA (blue, column 3), and expression of YFP to visualize the cytoplasm (green, column 4). The three fluorescent micrographs are overlaid in column 5. (A) Images of the SEA008 control strain in which σ^E^ was not inhibited are shown. (B–D) Images of SEA007 following σ^E^ inhibition, reveal blebs that contain YFP and stain with DAPI (B and C) and that contain YFP but do not stain with DAPI (D). In (D), the arrow marks a bleb lacking DAPI staining and the arrowhead marks a lysed cell that retained DAPI staining, but lost YFP. Scale bars are 2 µm. Over 1,000 cells were examined by fluorescence microscopy and typical micrographs are represented here. Scanning electron micrographs (E) and transmission electron micrographs (F) of SEA007 following σ^E^ inhibition. Scale bars are 1 µm. No blebs were seen on cells in control cultures.

We next examined the morphology of cells following inhibition of σ^E^. By 2 hours after addition of IPTG to overexpress *rseA* and *rseB* (the time that the cfu/ml began to drop), two phenotypes were evident by phase contrast microscopy. Ghosted cells and cells with blebs began to appear in the culture. The blebs formed primarily along the lateral wall of the cells and were found less frequently at either the poles or the septum. Usually only one bleb formed per cell. By 2.5 hours after addition of IPTG, approximately 20% of cells in the culture had blebs (Fig. 6) and the number of ghosted cells increased along with the amount of cellular debris, indicative of lysis. In a typical experiment, approximately 100 cells were viewed at each time point. The location of the blebs and the timing of their appearance with respect to loss of viability were very reproducible, suggesting that the phenotypes were related. By 3–4 hours after addition of IPTG some cells had additional blebs, the number of ghosted cells and the amount of cell debris increased, and many cells clumped together. Similar phenotypes were observed when σ^E^ was inhibited by proteolytic stabilization of RseA through depletion of the DegS or RseP proteases (data not shown) and were not found following overexpression of *rseA*D11H and *rseB* from pRseA^D11H^B, providing further evidence that the phenotypes were due to inhibition of σ^E^ and not overexpression of *rseA* and *rseB*. In addition, the same phenotypes were seen when the cells were grown in glucose or glycerol minimal media supplemented with amino acids indicating that the phenotypes were not a function of growth rate or medium composition.

To further probe the morphology of cells following inhibition of σ^E^, fluorescent probes were used to detect cytoplasm, nucleic acids, and membranes in live cells. We visualized the cytoplasm by expressing the soluble fluorescent protein, YFP, from an expression construct integrated in the chromosome. Membranes and nucleic acids were visualized respectively with the FM4-64 and DAPI fluorescent dyes, which were added directly to the cultures. Neither the dyes nor cytoplasmic expression of YFP altered the phenotypes associated with inhibition of σ^E^. All of the cells and blebs were outlined with FM4-64, and both the cells and the blebs contained YFP within the FM4-64 boundary, with the exception of the ghosted cells (Fig. 6A–D). The ghosted cells were outlined with FM4-64, but no longer contained YFP (Fig. 6D, indicated by the arrowhead). YFP is not proteolytically labile, therefore the lack of a YFP signal in the ghosted cells is likely to be due to release of cytoplasmic contents upon lysis. All of the cells had DAPI staining, and both the compaction of the nucleoids and the number of nucleoids per cell did not differ significantly from control cells, suggesting that loss of σ^E^ activity does not cause major defects in chromosome replication or segregation (Fig. 6A–D). Approximately 50% of the cells with blebs contained DAPI staining within the bleb, in addition to YFP (Fig. 6B,C). We hypothesize that some blebs do not contain DAPI staining (Fig. 6D, indicated by the arrow), either because the nucleoid was simply not drawn into the blebs, or there were not enough nucleic acids present to be visualized with DAPI.

Electron microscopy was employed to determine if there were any additional changes in cell morphology not detectable with light microscopy. Scanning electron micrographs revealed no significant alterations other than cells with blebs and ghosted cells (Fig. 6E). The surface of the cells with blebs and ghosted cells were similar to those of cells from control cultures without overexpression of *rseA* and *rseB*. Using transmission electron microscopy (TEM) we obtained several images containing sections through a bleb. In these images, the inner and outer membranes could be clearly traced around the bleb, and cytoplasmic material was present in the bleb (Fig. 6F). No evidence of outer membrane vesiculation, which has been associated with alterations in σ^E^ activity [33], [34], or other gross morphological defects were observed in the TEM or SEM images.

### Isolation of multicopy suppressors of the requirement of σ^E^ activity for viability

The phenotypic studies demonstrate that when σ^E^ activity is depleted, cell envelope integrity is compromised, resulting in lysis and bleb formation. However, these phenotypes are not readily explained by loss of expression of any gene(s) in the σ^E^ regulon (see Discussion). To gain additional information about why σ^E^ is essential, we turned to genetics and isolated multicopy suppressors that allowed *E. coli* to live in the absence of σ^E^ activity. Cells containing pRseAB (strain SEA007) were transformed with pools of plasmids from the ASKA ORF library, in which each *E. coli* ORF was cloned under the control of an IPTG-inducible promoter [35]. Transformants were plated in the presence of IPTG to induce overexpression of *rseA* and *rseB* and the gene encoded on the ASKA plasmid. Of approximately 10,000 transformants, 44 putative suppressors were recovered.

Two classes of genes can be isolated with this selection, those encoding genes that restore σ^E^ activity and those that no longer require σ^E^ for growth. We distinguished between these classes using the chromosomally encoded σ^E^-dependent *lacZ* reporter gene (*rpoH*P3-*lacZ* [36]) in strain SEA007 and detected σ^E^ activity by plating in the presence of Xgal and IPTG. Of the 44 clones isolated in the selection, 26 formed blue colonies, indicating that overexpression of the gene on the plasmid restored σ^E^ activity, and 18 formed white colonies, indicating that σ^E^ activity was not restored and therefore was no longer required for growth. This latter class contained plasmids that were potential suppressors of the requirement of σ^E^ for viability. For each of these 18 plasmids, we verified that suppression was linked to the plasmid, the plating efficiencies when *rseA* and *rseB* were overexpressed were no longer reduced, and the morphological defects were significantly reduced.

The selection identified plasmids that permit growth in the presence of the overexpression of *rseA* and *rseB*. However, these strains could have low levels of σ^E^ activity that contribute to viability. We therefore determined whether the *rpoE* gene could be deleted when the potential suppressor genes were overexpressed using a cotransduction assay. A *nadB*::Tn*10* allele immediately upstream of a *rpoE*::*kan* deletion was introduced by P1 transduction into wild-type strains containing each of the 18 plasmids, and *nadB*::Tn*10* transductants were selected. Because the genes are tightly linked, the *rpoE*::*kan* allele will be cotransduced unless *rpoE* is essential (Table 2). The *rpoE* deletion was cotransduced in strains carrying 2 of the 18 plasmids (Table 2). For the remainder of the strains, the *rpoE* deletion was not cotransduced, indicating that the *rpoE* gene was still required for viability. The two multicopy suppressors of *rpoE* essentiality were *yhbW* and *ptsN* (Tables 1 and 2). *yhbW* encodes a putative luciferase-like monooxygenase of unknown function. *ptsN* encodes EIIA^Ntr^, a protein related to the enzyme IIA components of PTS carbohydrate transport systems [37]. However, EIIA^Ntr^ is not known to be associated with any known transporters in *E. coli* and the Ntr phosphoryl relay is instead thought to play a role in signaling [37]–[39]. *ptsN* is a member of the *rpoE* regulon [9] and we focused on it for further studies.

**Table 2:** Cotransduction of *nadB*::Tn*10* and *rpoE*::*kan*

recipient	plasmid^a^	*nadB*::Tn*10* b	*rpoE*::*kan* c
SEA001 (wild type)	―	159	1
CAG43113 (*sup+*)	―	184	184
SEA4270	pYhbW	48	48
SEA4254	pPtsN	153	153
SEA4106	pPtsNH73A	24	0
SEA4110	pPtsNH73A/H120A	24	0
SEA4230 (Δ*trkA*)	―	11	0
SEA4234 (Δ*trkA*)	pPtsN	23	23
SEA4144 (Δ*ptsP*)	―	20	0
SEA4156 (Δ*ptsP*)	pPtsN	12	12
SEA4143 (Δ*ptsO*)	―	12	0
SEA4155 (Δ*ptsO*)	pPtsN	15	15
SEA4131 (Δ*ptsN*)	―	24	0

a “―” denotes no plasmid

b number of colonies isolated by P1 transduction selecting for *nadB*::Tn*10*

c number of *nadB*::Tn*10* transductants shown to also have *rpoE*::*kan*

### Phosphorylation of EIIA^Ntr^ is required for suppression

Since EIIA^Ntr^ is part of a phosphoryl relay and the phosphorylation state of EIIA^Ntr^ is important for its activity [40], [41], we determined whether phosphorylation of EIIA^Ntr^ was important for suppression. EIIA^Ntr^ is phosphorylated at His73 and possibly His120 [41]. We constructed two variants, *ptsN*H73A and *ptsN*H73A/H120A, and tested them for their ability to suppress the requirement of σ^E^ for viability in the cotransduction assay. The *rpoE*::*kan* allele could not be cotransduced with *nadB*::Tn*10* (Table 2) suggesting that phosphorylation of EIIA^Ntr^ is required for survival in the absence of σ^E^. Overexpression of both EIIA^Ntr^ variants could be detected on Coomassie-stained protein gels indicating that lack of suppression was not owed to instability of the variant proteins (data not shown).

The *ptsP* and *ptsO* genes encode EI^Ntr^ and NPr, respectively, and form a phosphoryl relay that transfers a phosphate from phosphoenol pyruvate (PEP) to EI^Ntr^ to NPr to EIIA^Ntr^ [37], [39]. Since phosphorylation of EIIA^Ntr^ was required for suppression, we asked whether the other components of the phosphoryl relay were also required by determining whether overexpression of *ptsN* could still suppress the essentiality of *rpoE* in strains lacking *ptsP* or *ptsO*. These genes were not required for suppression by overexpressed *ptsN*; *nadB*::Tn*10 rpoE*::*kan* cotransductants were obtained in both strains (Table 2). Additionally, the *rpoE* gene could not be deleted in Δ*ptsN*, Δ*ptsO*, or Δ*ptsP* strains without overexpression of *ptsN*. Since the results with the point mutations in *ptsN* indicate that phosphorylation of EIIA^Ntr^ is required for suppression, it is likely that paralogous proteins from other phosphoryl relays might compensate for the loss of the Ntr components and phosphorylate EIIA^Ntr^ in their absence.

PtsN has been shown to bind to and inhibit potassium uptake by the potassium transporter TrkA [40]. K^+^ is the major cation in the *E. coli* cytoplasm and participates in a variety of processes related to known functions of σ^E^ including adaptation to osmotic stress and maintenance of turgor pressure [42]–[44]. In addition, K^+^ binds and regulates a number of intracellular enzymes including RNA polymerase (as potassium glutamate) [45]. Therefore, if overexpression of *ptsN* suppresses the requirement of σ^E^ for viability by inhibiting TrkA, *rpoE* should not be essential in a Δ*trkA* strain. However, when a Δ*trkA* strain was used as the recipient in the cotransduction assay, only *nadB*::Tn*10* transductants were obtained indicating that Δ*trkA* is not a suppressor (Table 2). Furthermore, the *trkA* gene was not required for suppression by *ptsN*, as cotransductants were readily obtained in a Δ*trkA* strain when *ptsN* was overexpressed (Table 2).

### Overexpression of *ptsN* lowers envelope stress

**Figure 7:**
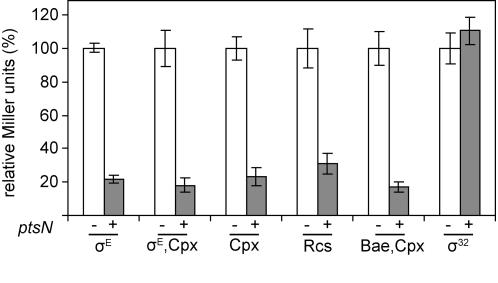
Overexpression of *ptsN* reduces cell envelope stress. β-galactosidase activity from the *rpoH*P3-*lacZ* (σ^E^, SEA4254), *degP*-*lacZ* (σ^E^ and Cpx, SEA4181), *cpxP*-*lacZ* (Cpx, SEA4179), *rprA*-*lacZ* (Rcs, SEA4199), *spy*-*lacZ* (Cpx and Bae, SEA4189), and *htpG*-*lacZ* (σ^32^, SEA4185) fusions was measured in overnight cultures, with and without overexpression of *ptsN*. Activity of each *lacZ* fusion was normalized to that of cultures with no *ptsN* overexpression and set to 100%. Average values and standard deviations from a minimum of 3 experiments are shown.

**Figure 8:**
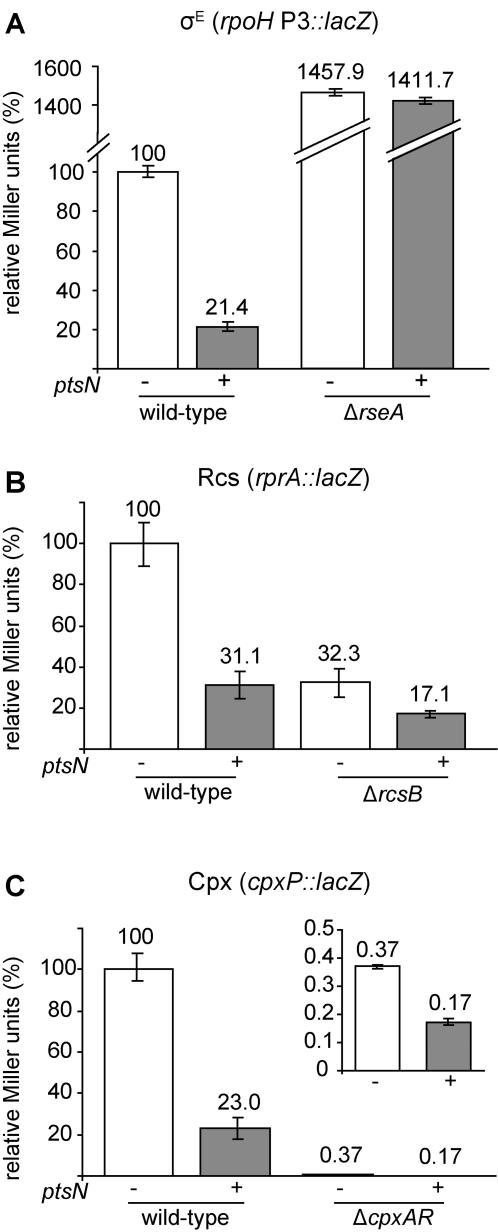
* ptsN* requires known regulators to reduce stress sensed by the σ^E^, Rcs, and Cpx envelope stress pathways. In each panel the β-galactosidase activity was measured with and without overexpression of *ptsN* in a WT strain and in a strain lacking known regulators of the respective stress responses. Measurements are expressed as the percentage of reporter gene activity in the WT strain without *ptsN* overexpression and this value is noted above the columns. Relative reporter gene activities are shown for (A) the *rpoH*P3-*lacZ* fusion in WT and Δ*rseA* strains (SEA4254 and SEA4228), (B) the *rprA*-*lacZ* fusion in WT and Δ*rcsB* strains (SEA4199 and SEA4203), and (C) the *cpxP*-*lacZ* fusion in WT and *cpxR*::Ω*spec* strains (SEA 4179 and SEA4251). The inset in C shows the *cpxR*::Ω*spec* strain with and without *ptsN* overexpression. The basal level of expression is >250-fold lower in *cpxR*::Ω*spec* cells than in WT cells and is not easily seen on the main plot.

The basal levels of several envelope stress responses are lower in a Δ*ydcQ* strain, the other characterized suppressor Δ*rpoE* lethality [10]. In addition, σ^E^-dependent *rpoH*P3-*lacZ* activity is low in a strain containing an unmapped Δ*rpoE* suppressor [46]. Consistent with these results, overexpression of *ptsN* lowered activity of the σ^E^-dependent *rpoH*P3-*lacZ* reporter in a wild-type *rpoE^+^* strain (Fig. 7 and 8A). To determine whether this effect was specific to σ^E^, or if *ptsN* overexpression affected other envelope stress responses, we measured the effects of *ptsN* overexpression on reporters for the following envelope stress responses (Fig. 3): Cpx (*cpxP*-*lacZ* reporter), σ^E^ and Cpx (*degP*-*lacZ* reporter), Bae and Cpx (*spy*-*lacZ* reporter), and Rcs (*rprA*-*lacZ* reporter). A strain carrying a reporter for the σ^32^-dependent cytoplasmic heat shock response (*htpG*-*lacZ*) was included to assay whether *ptsN* also lowered cytoplasmic stress (Fig. 3). Overexpression of *ptsN* significantly lowered expression of the *lacZ* fusions that are regulated by envelope stress response factors, but had no effect on the σ^32^-dependent cytoplasmic stress reporter (Fig. 7). The latter result indicates that overexpression of *ptsN* does not lower β-galactosidase activity *per se,* nor does it have a dampening effect on overall gene expression.

*ptsN* overexpression could reduce the basal levels of envelope stress that activate the stress signaling pathways or trigger a cytoplasmic signal that downregulates the effectors of the signaling pathways independently of cell envelope stress. If the latter idea is correct, then *ptsN* should lower reporter activity independently of the envelope signal transduction pathways. In a strain lacking the antisigma factor RseA, σ^E^ activity is high and unresponsive to known regulatory signals that originate in the cell envelope, but is still responsive to levels of the cytoplasmic signaling factor, ppGpp [24]. Overexpression of *ptsN* did not lower activity of the *rpoH*P3-*lacZ* reporter in a Δ*rseA* strain, suggesting that signal transduction through RseA is required for *ptsN* to lower σ^E^ activity (Fig. 8A).

If *ptsN* overexpression does in fact lower the basal level of envelope stress, then the decrease in reporter gene expression should be dependent on the transcriptional activators associated with the envelope stress pathways. Interestingly, in the Δ*ydcQ* strain the decrease in reporter gene expression of the Cpx-regulated *cpxR-lacZ* reporter was not dependent on the response regulator CpxR [10]. We measured the effects of overexpression of *ptsN* on the Cpx-dependent *cpxP-lacZ* and Rcs-dependent *rprA-lacZ* reporter fusions in strains lacking their respective transcriptional regulators, CpxR and RcsB. In a WT strain, overproduction of *ptsN* lowered expression of the *rprA-lacZ* reporter 3.2-fold compared with 1.8-fold in the Δ*rcsB* strain (Fig. 8B) and lowered expression of the *cpxP-lacZ* reporter 4.3-fold compared with 2.2-fold in the *cpxR*::Ω*spec* strain (Fig. 8C). It has been reported previously that the *cpxP-lacZ* reporter is not expressed in a Δ*cpxR* variant of the *E. coli* strain MC4100 [10], [47]. However, we were able to reproducibly measure β-galactosidase activity from this reporter in a *cpxR*::Ω*spec* variant of strain MG1655, although expression was quite low (∼250-fold lower than in a WT background). These results indicate that decreased activity of the reporters due to *ptsN* overexpression is at least partially dependent on *cpxR* and *rcsB*, suggesting that EIIA^Ntr^ lowers envelope stress sensed by both pathways.

## Discussion

Despite extensive knowledge about the regulatory pathways that control σ^E^ activity, the stresses that activate these pathways, and the genes that σ^E^ transcribes, the essential role of σ^E^ in the cell has remained elusive. In this work we demonstrate that σ^E^ is required for maintenance of cell envelope integrity. When σ^E^ activity was inhibited, envelope stress sensed by several stress responses increased, indicating that the cell envelope was impacted. Many cells lysed and some developed blebs containing cytoplasmic material along their sides. Interestingly, these phenotypes were not accompanied by major alterations in the composition of the envelope membranes. We identified two multicopy suppressors, *ptsN* and *yhbW*, that allowed cells to live in the absence of the *rpoE* gene. *ptsN* is a member of the σ^E^ regulon [9] and lowers the basal level of activity of several envelope stress responses by an unknown mechanism when overexpressed.

### How does loss of σ^E^ activity lead to bleb formation and lysis?

The blebs that form following inhibition of σ^E^ resemble bulges formed when *E. coli* are treated with penicillin-like antibiotics that inhibit peptidoglycan synthesis, or when an inactive variant of the peptidoglycan synthase PBP 1B is overproduced [48], [49]. These treatments are also lytic, and lysis is thought to be triggered by holes formed in the peptidoglycan layer by murein hydrolases acting in the absence of peptidoglycan synthesis [48], [49]. The similarity in the phenotypes suggests that inhibition of σ^E^ leads to weakening of and/or defects in the peptidoglycan layer. These defects potentially have two major outcomes. Either a hole forms in the peptidoglycan that is large enough to accommodate extrusion of the inner membrane and cytoplasm, evidenced by YFP signal in all of the blebs, or the cell lyses, evidenced by loss of YFP signal in the ghosted cells. The observation that blebs form in only 20% of the cells is likely to be due to the inherent stochastic nature of the process. Many cells may lyse before a bleb can form. Further support for the model that inhibition of σ^E^ leads to defects in the peptidoglycan layer comes from preliminary results with a strain lacking four enzymes involved in peptidoglycan synthesis, PBPs 4, 5, 6, and 7, which results in irregular peptidoglycan synthesis [50]. This strain develops blebs and lyses more quickly following inhibition of σ^E^ than the isogenic WT strain (data not shown). If the peptidoglycan is affected, we should be able to reduce cell death by growing the cells in high osmolarity medium. Unfortunately, this experiment was not technically feasible. High osmolarity induced the σ^E^ response such that σ^E^ was no longer inhibited by RseA, even when RseA was overexpressed (data not shown).

Even though these phenotypes are consistent with the model that loss of σ^E^ activity leads to alterations in the peptidoglycan layer resulting in bleb formation and lysis, we cannot eliminate the possibility that bleb formation is caused by a different event, such as aberrant cell division. Future experiments examining the integrity of the peptidoglycan layer in more detail and the localization of cell division proteins following inhibition of σ^E^ should help distinguish between these possibilities.

### Relationship between the phenotypes and the known functions of σ^E^ regulon members

Because the only reported function of σ^E^ is to direct transcription, altered regulation of one or more of the genes in the σ^E^ regulon should ultimately lead to bleb formation and loss of viability when σ^E^ activity is blocked. However, given the known functions of σ^E^ regulon members, we cannot point to one specific gene or set of genes in the regulon that is obviously associated with these phenotypes. Depletion phenotypes have been characterized in detail for five essential σ^E^-regulated genes (*yaeT*, *yfiO*, *imp*, *lptA* and *lptB*), which encode components of complexes that assemble LPS or OMPs in the outer membrane [28], [51]–[54]. Depletion of these genes causes striking defects in the cell envelope, but not those observed following inhibition of σ^E^ [28], [51]–[55].

The blebbing phenotype suggests that peptidoglycan or cell division is affected by loss of σ^E^. Although σ^E^ does not transcribe any known murein hydrolases or biosynthetic enzymes directly involved in peptidoglycan synthesis, it does transcribe *bacA*, which encodes an undecaprenyl pyrophosphate phosphatase [9], [56]. BacA hydrolyzes a phosphate moiety from undecaprenyl pyrophosphate to generate undecaprenyl phosphate, which is a lipid carrier required for synthesis of peptidoglycan and other cell wall polymers [56]. However, *E. coli* possesses at least three additional undecaprenyl pyrophosphate phosphatase enzymes and cells lacking *bacA* alone have no obvious morphological phenotypes [56]. A σ^E^-dependent promoter is also found upstream of the cell division protein FtsZ [9], but this promoter is one of multiple promoters that contribute to the complex regulation of *ftsZ* expression [57], making it unlikely that loss of σ^E^ activity would have a large effect under non-stressed growth conditions.

Since no direct connection can yet be made convincingly between σ^E^ regulon members and the observed phenotypes, we think it likely that the phenotypes are caused by indirect effects resulting from loss of or altered regulation of σ^E^-dependent genes. For example, σ^E^ transcribes genes required for synthesis of the lipid A component of LPS, *lpxA*, *lpxB*, and *lpxD* [9]. LpxA catalyzes the first step in lipid A synthesis and uses the same substrate, UDP-*N*-acetyl-D-glucosamine, as is used in the first dedicated step of peptidoglycan biosynthesis [58]. Changes in *lpxA* and *lpxD* expression in the absence of σ^E^ could alter the flux of substrates through these pathways, affecting peptidoglycan synthesis and the integrity of the peptidoglycan layer.

### Suppressors of Δ*rpoE* lethality

In this work two genes were identified whose overexpression suppresses both the loss of viability following inhibition of σ^E^ and the requirement of the *rpoE* gene for viability. Of the three known suppressors of Δ*rpoE* lethality (Δ*ydcQ* [10] and overexpression of *ptsN* and *yhbW*), the only gene with any known roles in the cell is *ptsN*. *ptsN* is a member of the σ^E^ regulon, which suggests that it plays a role in cell envelope processes [9]. Several functions have been associated with the nitrogen PTS system, of which *ptsN* is a member, including balancing nitrogen and carbon metabolism, suppression of a temperature-sensitive allele of the essential GTPase Era by a Δ*ptsN* mutant, and inhibition of the potassium transporter TrkA [37], [38], [40]. This latter role of *ptsN* held the most promise for providing insights into the mechanism of suppression because K^+^ is important for osmotic regulation and can regulate RNA polymerase, all activities associated with σ^E^ [42], [44], [45]. However, we demonstrated that *trkA* is not required for suppression by *ptsN*, nor is Δ*trkA* a suppressor, indicating that EIIA^Ntr^ has additional functions, yet to be discovered, that are responsible for suppression of Δ*rpoE* lethality. As discussed below, our results indicate that this novel activity likely involves regulation of cell envelope stress responses.

### Lowered envelope stress responses, a characteristic of σ^E^ suppressors

The basal level of activity of multiple envelope stress responses is reduced in the presence of all σ^E^ suppressors for which data is available, overexpression of *ptsN*, Δ*ydcQ* [10] and an uncharacterized suppressor [46], suggesting that lower activity of envelope stress responses is an important part of the suppression mechanism. Preliminary results indicate that the basal levels of cell envelope stress responses are also lower following overexpression of *yhbW*, the other multicopy suppressor that we isolated. In light of these results, one model for suppression is that the suppressors strengthen the cell envelope such that loss of σ^E^ activity and the resultant impact on the cell envelope are no longer lethal. If this model is correct, then the suppressors should lower envelope stress sensed by the signal transduction pathways that monitor the cell envelope. We found that the effects of *ptsN* overexpression on reporter gene expression for the Rcs and Cpx pathways were at least partially dependent on their respective stress regulators. In addition, σ^E^ activity was not altered by *ptsN* overexpression in a strain lacking *rseA*, the only known envelope sensor for the pathway. Although it is possible that EIIA^Ntr^ was unable to overcome the high level of activation of σ^E^ in a Δ*rseA* strain background, we think it unlikely based on observations with another regulator of σ^E^, which does not act through RseA. σ^E^ activity can be both activated and reduced in a Δ*rseA* strain in response to the cytoplasmic regulator ppGpp [24], suggesting that the effects of *ptsN* overexpression should be discernible. In contrast, the reduction in activity of the Cpx reporter gene examined in the Δ*ydcQ* strain was not dependent on *cpxR* [10]. However, epistasis experiments were only reported for the one reporter, and it is not known whether the activity of other cell envelope stress responses were reduced in the Δ*ydcQ* background independently of their respective signal transduction pathways [10].

### Why is σ^E^ essential in *E. coli*?

The functions of core members of the σ^E^ regulon are conserved in a number of bacterial species [9]. However, σ^E^ is not essential in many of these bacteria including closely related species such as *Salmonella typhimurium* [59]. This observation led, in part, to a model proposing that σ^E^ is not essential because it is required to transcribe a particular gene or set of genes [10]. Instead, σ^E^ is essential because the bacteria overreact to its absence and, through an unknown mechanism that requires the *ydcQ* gene product, activate an unknown cell death pathway that kills the cells [10]. Our data are consistent with this model. However, we suggest that even though the functions of core regulon members are conserved in bacterial species in which σ^E^ is not essential [9], significant differences can exist in the properties of the cell envelopes of different species and even strains within a species that may determine whether the bacteria can survive in the absence of σ^E^. For example, the majority of the work on σ^E^, including that presented in this paper, has been performed in K12 strains of *E. coli*, which lack the O-antigen of LPS [60]. This variation could significantly alter the properties of the bacterial cell envelope, making the bacteria more sensitive to disruptions in cell envelope integrity so that σ^E^ would be essential. Therefore, we propose that σ^E^ is indeed essential because it is required to transcribe genes in its regulon. In the absence of σ^E^ these genes are not properly regulated, leading to disruption of cell envelope integrity and lysis. Future experiments delving in greater depth into the phenotypes of cells lacking σ^E^, the roles of σ^E^ regulon members of unknown function, and the mechanism of suppression should help to distinguish between these models and provide new insights into the role of this important cell envelope stress response.

## Materials and Methods

### Media, strains, and plasmids

Strains are derivatives of MG1655, unless otherwise noted, and are listed in Table 3. Cultures were grown in Luria Bertani (LB) broth at 30°C with shaking in a gyrotary water bath. Antibiotics were used at the following concentrations: ampicillin (amp) 100 µg/ml, kanamycin (kan) 30 µg/ml, tetracyline (tet) 10 µg/ml, spectinomycin (spec) 50 µg/ml, and chloramphenicol (cam) 20 µg/ml. Altered alleles and the stress response reporters were transferred into the appropriate recipient strains by transduction with P1_vir_ according to standard methods [61], with the exception that sodium citrate was not used because it can be toxic to cells with compromised membranes. The *rseA*D11H mutation was made in the pRseAB plasmid and the *ptsN* H73A and H73A/H12A mutations in the pPtsN plasmid using the QuikChange site-directed mutagenesis kit (Stratagene) and verified by sequencing. The *ptsN*::*kan*, *ptsO*::*kan*, *ptsP*::*kan*, and *trkA*::*kan* alleles were obtained from the Keio collection [62] and moved by P1 transduction into SEA001. The *kan* allele was then removed with FLP recombinase by the method of Datsenko and Wanner [63] to create strains SEA4131, SEA4143, SEA4144, and SEA4230.

**Table 3:** Strains and Plasmids

Strain	Genotype	Reference
CAG43113	MC1061 *sup+*, same as CAG41001	[20]
CAG45146	MG1655 Δ*lacX74* Φλ[*htpG* P1-*lacZ*]	[65]
DH300	MG1655 Δ(*argF-lac*)*U169*; *rprA142-lacZ*	[66]
SEA001	MG1655 Δ*lacX74* Φλ[*rpoH*P3-*lacZ*]	[67]
SEA007	SEA001 pRseAB	[24]
SEA008	SEA001 pTrc99a	this work
SEA2000	SEA001 *nadB*::Tn*10* Δ*rseA*	[24]
SEA4041	CAG43113 *rpoE*::*kan*	this work
SEA4044	SEA001 pRseA^D11H^B	this work
SEA4106	SEA001 pPtsNH73A	this work
SEA4110	SEA001 pPtsNH73A/H120A	this work
SEA4114	SEA4041 *nadB*::Tn*10*	this work
SEA4131	SEA001 Δ*ptsN*	this work
SEA4143	SEA001 Δ*ptsO*	this work
SEA4144	SEA001 Δ*ptsP*	this work
SEA4155	SEA4143 pPtsN	this work
SEA4156	SEA4144 pPtsN	this work
SEA4177	MG1655 Δ*lacX74* λRS88[*cpxP*-*lacZ*] pRseAB	this work, reporter from [47]
SEA4179	MG1655 Δ*lacX74* λRS88[*cpxP*-*lacZ*] pPtsN	this work, reporter from [47]
SEA4181	MG1655 Δ*lacX74* λRS88[*degP*-*lacZ*] pPtsN	this work, reporter from [68]
SEA4183	CAG45146 pRseAB	this work
SEA4185	CAG45146 pPtsN	this work
SEA4187	MG1655 Δ*lacX74* λRS88[*spy*-*lacZ*] pRseAB	this work, reporter from [69]
SEA4189	MG1655 Δ*lacX74* λRS88[*spy-lacZ*] pPtsN	this work, reporter from [69]
SEA4190	DH300 pRseAB	this work
SEA4199	DH300 pPtsN	this work
SEA4203	4199 *rcsB*::*kan*	this work, *rcsB*::*kan* from [66]
SEA4204	SEA4181 *nadB*::Tn*10* Δ*rseA* pPtsN	this work
SEA4228	SEA2000 pPtsN	this work
SEA4230	SEA001 Δ*trkA*	this work
SEA4234	SEA4230 pPtsN	this work
SEA4251	SEA4179 *cpxR*::Ω*spec*	this work, *cpxR*::Ω*spec* from [68]
SEA4254	SEA001 pPtsN	this work
SEA4256	SEA007 pPtsN	this work
SEA4270	SEA001 pYhbW	this work
SEA4271	SEA007 pYhbW	this work
SEA4287	SEA007 Δ*(xylAxylB*)::*tet* ^R^ *tetA-yfp*	this work, *yfp* allele from Goulian lab collection
SEA4288	SEA008 Δ*(xylAxylB*)::*tet* ^R^ *tetA-yfp*	this work, *yfp* allele from Goulian lab collection
**Plasmids**		
pTrc99a	vector, pBR322 ori Amp^R^	Pharmacia
pRseAB	*rseA* and *rseB* in pTrc99a, same as pLC253	[15]
pRseA^D11H^B	RseAD11H in pRseAB	this work
pPtsN	*ptsN* in pCA24N, ASKA plasmid , Cam^R^	this work and [35]
pPtsNH73A	PtsNH73A in pPtsN	this work
pPtsNH73A/H120A	PtsNH73A/H120A in pPtsN	this work
pYhbW	*yhbW* in pCA24N, ASKA plasmid , Cam^r^	this work and [35]

### β-galactosidase assays

β-galactosidase assays were performed largely as described [24]. Overnight cultures were diluted to OD_600_ of 0.02 and grown with aeration at 30°C. *rseA* and *rseB* overexpression was induced by the addition of IPTG to 1 mM when cultures reached an OD_600_ of 0.1–0.2 for experiments shown in Figs. 2 and 4. In Figs. 7 and 8, β-galactosidase activity was determined from cultures grown for 12–14 hours with and without 1mM IPTG. Experiments with exponential phase cultures yielded similar results. A minimum of three independent experiments were performed for each strain and condition.

### Electron microscopy

Cells of SEA007 and SEA008 to be used for electron microscopy were grown in LB to an OD_600_ of 0.1–0.2, at which point IPTG was added to induce *rseA* and *rseB* overexpression in SEA007. Cells were harvested 2.5 hours later by centrifugation. Samples for scanning electron microscopy (SEM) were subjected to a primary fixation step in 6.25% glutaraldehyde in Sorenson's Buffer for 1.5 hours at room temperature then washed in 0.1 M cacodylate buffer. A secondary fixation step was performed by treating the samples with 2% osmium tetroxide in 0.1 M cacodylate buffer, followed by washing as before and dehydration in ethanol. Samples were dried, sputter coated and imaged on a JEOL JSM 5400 scanning electron microscope with a PGT Prism light element detector.

Samples for transmission electron microscopy (TEM) were prepared by cryo-fixation and freeze substitution. The samples were washed in 0.1 M cacodylate buffer and incubated in 18% glycerol (v/v) in 0.1 M cacodylate for 20 minutes at room temperature for cryo-protection. The sample was pelleted by centrifugation and 10 µl of the pellet was blotted onto filter paper and frozen in liquid nitrogen. Freeze substitution was carried out in 2% osmium tetroxide, 0.1% uranyl acetate in acetone at −90°C for 3 days. Samples were slowly warmed at −60°C for 14 hours, −30°C for 14 hours, and then 0°C for 1 hour. Samples were transferred to acetone, brought to room temperature, and then infiltrated with resin. Ultrathin sections were obtained using a Reichart-Jung Ultracut E microtome with a diamond knife. Sections were mounted on hexagonal mesh or open slot copper grids and stained with 2% uranyl acetate in 50% ethanol for 5 minutes followed by Reynold's lead stain for 2 minutes and washed with water. After drying, the grids were visualized on a JEOL JEM 1200 EXII transmission electron microscope. Images were taken with a digital TEITZ camera.

### Fluorescence microscopy

For fluorescence microscopy, location of the cytoplasm was visualized in strains SEA4287 and SEA4288, which express soluble YFP throughout the cytoplasm, but not the periplasm. Overnight cultures grown in LB/Amp were subcultured into media containing 1 ng/ml anhydrous tetracycline, which induces expression of YFP. IPTG was added when cells reached an OD_600_ of 0.1–0.2 and samples were taken for microscopy between 1.5 and 3.5 hours later. FM4-64 (Molecular Probes T-3166) and 4,6-diaminidino-2-phenylindole, DAPI, were added directly to samples at respective final concentrations of 5 µg/ml and 1 µg/mL. Cells were mounted on freshly prepared polylysine coated coverslips and visualized using an Olympus BX-61 epi-fluorescent microscope with a 100× phase oil objective (NA of 1.3), using an Olympus 41028 filter cube for YFP, Olympus 31000v2 filter cube for DAPI, and an Olympus 41001 filter cube for FM4-64. Images were captured with a Hamamatsu Orca-ER camera using Slidebook 4.1 software (Compix, Inc.). Deconvolution was performed using the AutoQuant software package (Media Cybernetics). Generally, less than 10 iterations were necessary to deconvolve images.

### Sucrose density gradient fractionation

Cultures of SEA007 were grown to an OD_600_ of 0.1 at which point IPTG was added to 1 mM to one culture to induce *rseA* and *rseB* overexpression. After 2.5 hours of growth, cells were harvested by centrifugation, resuspended in 10 mM HEPES pH 7.8, and lysed by French press. Discontinuous sucrose density gradients were prepared by layering 860 µl of 50%, 45%, 40%, 35%, and 30% sucrose solutions over a 200 µl 55% sucrose cushion. Lysates from equal numbers of cells for treated and control cultures were loaded onto the gradients, based on the OD_600_ of the cultures at the time of sampling. After centrifugation at 50,000 rpm for 15 hours, 150 µl fractions were collected from the top of the gradients. The specific gravity of each fraction was determined by measuring the refractive index. β-NADH oxidase activity of each fraction was determined as previously reported [64]. Total protein content was analyzed on 12% SDS-polyacrylamide gels with Coomassie blue staining. Western blotting was performed with polyclonal antibodies to the outer membrane protein FepA (gift of Kathleen Postle) and inner membrane protein FtsH (gift of Christophe Herman) and detected with horseradish peroxidase-conjugated donkey anti-rabbit IgG (GE Healthcare) using the ECL Plus kit (GE Healthcare).

### Genetic selections

A genetic selection was carried out to identify genes whose overexpression allowed cells to live when σ^E^ activity was inhibited. Strain SEA007 (carrying pRseAB) was transformed by electroporation with 5 pools of plasmids from the ASKA plasmid library [35], and the cells were plated on LB agar with 1 mM IPTG, Amp (to select for pRseAB), and Cam (to select for the library plasmids). The ASKA plasmid library (without the GFP tag) used in these studies consists of plasmids containing *E. coli* ORF's cloned under the control of an IPTG-inducible promoter [35]. Colonies that grew on IPTG were streaked again on LB/IPTG/Amp/Cam to confirm growth. Plasmids were then isolated, and library plasmids purified by transformation into DH5α with selection on LB/Cam. The selected ASKA plasmids were then transformed back into SEA007 to verify that they conferred growth on IPTG. Cells were also grown on plates containing Xgal to determine whether σ^E^ activity was restored (blue colonies) or was still inhibited (white colonies). The selected genes encoded on the ASKA plasmids were identified by sequencing.

### Determination of the essentiality of *rpoE*

The *nadB*::Tn*10 rpoE*::*kan* linked marker construct used in cotransduction assays was made in a strain that contains an unidentified suppressor of Δ*rpoE* (*sup*, CAG43113). The *rpoE* gene was deleted first, using the method of Datsenko and Wanner [63] to generate strain SEA4041. The *nadB*::Tn*10* insertion from SEA2000 was then moved into the *rpoE*::*kan* strain by P1 transduction, selecting for resistance to Kan and Tet to generate strain SEA4114. Transductants were verified by PCR to ensure that the *kan* allele was present and the *rpoE* gene was absent. The essentiality of *rpoE* was determined by assessing the cotransduction frequency of *nadB*::Tn*10* and *rpoE*::*kan* in recipient strains. The markers are tightly linked, so *nadB*::Tn*10* should be transduced independently only in strains in which *rpoE* is essential. Plasmids that suppressed lethality due to overexpression of *rseA* and *rseB* without restoring σ^E^ activity were transformed into SEA001. The resulting strains were transduced with a P1 lysate from SEA4114. Colonies were first plated on LB/Tet/IPTG to select for *nadB*::Tn*10* and to overexpress the putative suppressor gene on the plasmid. Transductants were then streaked on LB/Kan/IPTG to determine if *rpoE* had been deleted. PCR was used to verify the presence of the *nadB*::Tn*10* and *rpoE*::*kan* alleles and the absence of the *rpoE* gene. Deletion of the *rpoE* gene was also verified by western blotting with polyclonal antibodies specific to σ^E^. In each cotransduction experiment, SEA001 and CAG43113 were used as negative and positive controls, respectively.
